# Predicting functional impairment trajectories in amyotrophic lateral sclerosis: a probabilistic, multifactorial model of disease progression

**DOI:** 10.1007/s00415-022-11022-0

**Published:** 2022-03-10

**Authors:** Erica Tavazzi, Sebastian Daberdaku, Alessandro Zandonà, Rosario Vasta, Beatrice Nefussy, Christian Lunetta, Gabriele Mora, Jessica Mandrioli, Enrico Grisan, Claudia Tarlarini, Andrea Calvo, Cristina Moglia, Vivian Drory, Marc Gotkine, Adriano Chiò, Barbara Di Camillo, For the Piemonte, Valle d’Aosta Register for ALS (PARALS), for the Emilia Romagna Registry for ALS (ERRALS), A. Chiò, Rita Levi Montalcini, A. Calvo, C. Moglia, A. Canosa, U. Manera, R. Vasta, F. Palumbo, A. Bombaci, M. Grassano, M. Brunetti, F. Casale, G. Fuda, P. Salomone, B. Iazzolino, L. Peotta, P. Cugnasco, G. De Marco, M. C. Torrieri, S. Gallone, M. Barberis, L. Sbaiz, S. Gentile, A. Mauro, L. Mazzini, F. Marchi, L. Corrado, S. D’Alfonso, A. Bertolotto, M. Gionco, D. Leotta, E. Oddenino, R. Cavallo, M. De Mattei, G. Gusmaroli, C. Comi, C. Labate, F. Poglio, L. Ruiz, D. Ferrandi, L. Testa, E. Rota, M. Aguggia, N. Di Vito, P. Meineri, P. Ghiglione, N. Launaro, M. Dotta, A. Sapio, M. Giovanni, J. Mandrioli, J. Mandrioli, N. Fini, I. Martinelli, E. Zucchi, G. Gianferrari, C. Simonini, M. Vinceti, S. Meletti, V. Vacchiano, R. Liguori, Fabrizio Salvi, Ilaria Bartolomei, Roberto Michelucci, P. Cortelli, A. M. Borghi, A. Zini, R. Rinaldi, P. Cortelli, E. Sette, V. Tugnoli, M. Pugliatti, E. Canali, L. Codeluppi, F. Valzania, L. Zinno, G. Pavesi, D. Medici, G. Pilurzi, E. Terlizzi, D. Guidetti, S. Pasqua, M. Santangelo, M. Bracaglia, P. DeMassis, M. Casmiro, P. Querzani, S. Morresi, M. Longoni, A. Patuelli, S. Malagù, M. Longoni, M. Currò Dossi, S. Vidale

**Affiliations:** 1 Department of Information Engineering, University of Padova, Padua, Italy; 2 Department of Neuroscience, University of Torino, “Rita Levi Montalcini”, Turin, Italy; 3 Tel Aviv Sourasky Medical Center, Tel Aviv, Israel; 4 Centro Clinico Nemo Milano, Fondazione Serena Onlus, Milan, Italy; 5 Istituti Clinici Scientifici Maugeri IRCCS, Milan, Italy; 6 Azienda Ospedaliero Universitaria di Modena, Modena, Italy; 7 School of Engineering, London South Bank University, London, UK; 8 Hadassah University Hospital Medical Center, Jerusalem, Israel; 9 Department of Comparative Biomedicine and Food Science, University of Padova, Via Gradenigo 6/B, 35131 Padua, Italy

**Keywords:** Amyotrophic lateral sclerosis, Clinical trajectories, Prognosis modelling, Population model, Artificial intelligence, Dynamic Bayesian Networks

## Abstract

**Objective:**

To employ Artificial Intelligence to model, predict and simulate the amyotrophic lateral sclerosis (ALS) progression over time in terms of variable interactions, functional impairments, and survival.

**Methods:**

We employed demographic and clinical variables, including functional scores and the utilisation of support interventions, of 3940 ALS patients from four Italian and two Israeli registers to develop a new approach based on Dynamic Bayesian Networks (DBNs) that models the ALS evolution over time, in two distinct scenarios of variable availability. The method allows to simulate patients’ disease trajectories and predict the probability of functional impairment and survival at different time points.

**Results:**

DBNs explicitly represent the relationships between the variables and the pathways along which they influence the disease progression. Several notable inter-dependencies were identified and validated by comparison with literature. Moreover, the implemented tool allows the assessment of the effect of different markers on the disease course, reproducing the probabilistically expected clinical progressions. The tool shows high concordance in terms of predicted and real prognosis, assessed as time to functional impairments and survival (integral of the AU-ROC in the first 36 months between 0.80–0.93 and 0.84–0.89 for the two scenarios, respectively).

**Conclusions:**

Provided only with measurements commonly collected during the first visit, our models can predict time to the loss of independence in walking, breathing, swallowing, communicating, and survival and it can be used to generate in silico patient cohorts with specific characteristics. Our tool provides a comprehensive framework to support physicians in treatment planning and clinical decision-making.

**Supplementary Information:**

The online version contains supplementary material available at 10.1007/s00415-022-11022-0.

## Introduction

Amyotrophic Lateral Sclerosis (ALS) is a fatal neurodegenerative disorder causing progressive paralysis and usually leading to death within 2–4 years from symptom onset due to respiratory failure [1]. Despite relative uniformity during late disease stages, the phenotype at onset and earlier stages is highly variable [2]. Region of onset, relative involvement of upper or lower motor neurons, and progression rate can differ substantially between patients, even in those with a similar genetic aetiology [3]. Moreover, a variety of non-motor symptoms can be associated with motor impairment, with frontotemporal dementia (FTD) being the most common [4].

In addition to progressive disability, people with ALS and their caregivers are faced with uncertainty regarding the sequence and timing of future impairments. Clinicians also need tools to predict the timing of future interventions, and accurate predictive models will be critical in improving the efficiency of therapeutic trials. Finally, a stratification of ALS patients based on their pattern of progression could give hints on different mechanisms acting in disease pathogenesis and help clinical trial design.

Artificial intelligence (AI) and machine learning methods can be used to describe the disease process and to make predictions that are applicable to a wide range of patients, as well as to develop personalised approaches to care tailored to the patients’ characteristics. So far, different predictive models of ALS progression have been developed, with the main goals being the prediction of future progression [5–10], and stratification of the patients into meaningful subgroups [11–13]. With respect to the predictive models, among the main considered outcomes there are ALS progression, change in weight, respiratory insufficiency, and survival [7]. Many of these models were developed using data from the Pooled Resource Open-Access ALS Clinical Trials (PRO-ACT) [14]. On one hand, PRO-ACT represents an invaluable resource for research studies on ALS, since its large sample size and visits’ frequency guarantee statistically significant analyses and allows a good disease progression characterization. Nonetheless, clinical trial cohorts are not fully representative of the general ALS population and their follow-up is limited to trials’ duration [15].

To overcome this limitation, some models developed on PRO-ACT were validated on external clinical cohorts, as in Taylor et al. [6]. Other models were directly developed on clinical cohorts [8, 16], or clinical datasets were integrated with the PRO-ACT dataset [12, 17].

From a general point of view, however, practical use of AI in health management is still limited. This may be due to a number of reasons such as lack of model interpretability and usability in different scenarios. For example, while being useful for predicting single survival or intervention endpoints (or a related risk score), prognostic models available in the literature have a limited ability to give a global vision of the disease evolution over time, including the progression of different intercorrelated variables and the management of patients' clinical heterogeneity.

Focusing more specifically on the needs related to this disease, in ALS patients need support to deal with an increasing need of care at home, alternated to periods in hospitals. Moreover, they experience a constant uncertainty regarding the timing of the impairments associated with the disease and face a considerable psychological and economic burden that also involves their caregivers. Clinicians, on the other hand, need tools able to support them in a multifactorial view of disease progression able to highlight the interplay of numerous multidimensional factors.

Based on these considerations, the aim of this work was to develop a model of disease progression able to predict the ALS main functional impairments in walking/self-care, breathing, swallowing and communicating, and, in addition, patients’ survival, based on the dynamic Bayesian network (DBN) approach. DBNs allow to generate, on the one hand, a graph showing how the variables influence each other over time and, on the other, the trajectories of progression of the disease, which show how the probability of death or functional impairment in the 4 domains mentioned above varies over time. The model, which was developed employing data from different international clinical centres, can be used to simulate ALS progression starting from the individual data of a specific patient at a specific visit, thus allowing to follow the probabilistic evolution of the disease in a population with the same characteristics. It also allows generating and comparing in silico cohorts of patients characterised by specific phenotypes, *e.g.*, bulbar vs. spinal onset, allowing the visualisation of different temporal phenotypes of disease evolution and the investigation of the effect of specific risk factors on the progression.

## Materials and methods

This work was performed in the context of the *CompALS* project, an Italian-Israeli collaboration. The study was approved by the ethical committees of the coordinating and participating centres. Written informed consent to participate in the study was obtained from all the patients or their legal representatives. The databases were anonymised according to the privacy protection legislation of Italy and Israel. The data used for training and validation of algorithms in this study are available upon reasonable request to the different centres involved in the study.

### Participants

ALS patients were recruited from two population-based registers, the Piemonte and Valle d'Aosta ALS register (PARALS) [18] and the Emilia-Romagna ALS register (ERRALS) [19], and four tertiary ALS clinics: Tel Aviv Medical Center, Hadassah University Hospital Medical Center (Jerusalem), Nemo Clinical Center (Milan), and Salvatore Maugeri Foundation (Milan). ALS diagnosis was assessed according to the El Escorial revised criteria [20].

### Data collection

For each patient, several demographic and clinical characteristics were considered. To depict different cases of use based on the available clinical variables, we aggregated the available demographic and clinical information into two datasets, and developed two distinct versions of the tool.

The first dataset (named “ITIS” in the following), includes the more frequently available prognostic variables from all the six Italian and Israeli data sources: sex, onset site, age at onset, diagnostic delay, and the revised ALS Functional Rating Scale (ALSFRS-R) scores [21], together with the survival information (time from ALS onset to either tracheostomy/death, or censoring information). This dataset represents a sort of basic scenario.

The second dataset (named “IT” in the following), comprises only data from the Italian registers/centres and includes a wider set of variables, thus representing a more advanced scenario with a higher level of detail on patient’s characterization. With respect to ITIS, it additionally includes features recognised as potentially prognostic in the scientific literature, such as genetic mutations (genes C9orf72, FUS, SOD1 and TARDBP), ALS family history, presence of frontotemporal dementia (FTD) detected through neuropsychological testing, premorbid body mass index (BMI) and BMI at diagnosis, forced vital capacity (FVC) at diagnosis, and the utilisation of respiratory (non-invasive ventilation, NIV) and nutritional (percutaneous endoscopic gastrostomy, PEG) supports.

In both datasets, starting from the visit times we derived two additional temporal variables: time between visits, TBV, and time since onset, TSO. These variables allow to account for different observation windows and different data sampling time among subjects, as well as to explicitly model the variation of the visit frequency as the disease progresses.

For both the ITIS and the IT datasets, the tool was developed on a dataset (named training set in the following, according to the machine learning habit) and was validated on a completely independent corresponding set of data (named test set in the following).

### Functional impairment assessment

To model the disease progression in terms of subjects’ functional impairments in walking/self-care, breathing, swallowing and communicating, we converted the ALSFRS-R scores into the Milano-Torino staging (MiToS) system [22], obtaining 4 dynamic variables that switch from 0 to 1 when a specific functional domain is impaired. These four MiToS stages were used in the model as functional outcomes to quantitatively characterise the evolution of the disease over time, together with the survival. For further considerations on the ALS staging systems see Section “1. ALS staging systems” in the Supplementary Information.

### Dynamic Bayesian network model

As a modelling technique, we used the dynamic Bayesian networks (DBNs) [23]. DBNs are computational models that encode the conditional dependence relationships among the variables of a multivariate dataset over time. They provide an explicit representation of the variable set and their inter-dependencies, as obtained from clinical data and domain knowledge: graphically, they are represented as directed acyclic graphs with nodes representing the variables, and directed edges representing the conditional dependence over subsequent time steps of a node (child) from one or more others (parents). DBNs are well suited for describing the evolution of diseases [24–26], since they provide an explicit representation of the variable set and their inter-dependencies, as well as the means to learn not only from the data but also from domain literature and expert knowledge. In the learning phase, a DBN uses the entire sequence of visits of the training set’s patients. Specifically, by looking at all the couples of consecutive visits at time (*t −* 1) and (*t*) for all the training patients, the DBN computes the conditional probability of each variable at time (*t*) given the values of its parents at time (*t − *1). Once a DBN model is learned, it can be used to interpret the relationships among variables, to predict and simulate disease progression in in silico populations or more specific sub-cohorts of patients, and to evaluate the effects of specific risk-factors on disease prognosis.

Here, stemming from our preliminary methodological work on the PRO-ACT database [27], we learned the DBNs in turn from the ITIS and IT training sets using *bnstruct* [28], an R package that performs structure and parameter learning on discrete/categorical data over a discrete number of time steps. First, we discretised the continuous variables according to their distribution quantiles in the training sets (for the thresholds used for the quantisation, see Section “2. Datasets” in the Supplementary Information). Then, we learned the DBNs on the training sets using the Max–Min Hill-Climbing algorithm (MMHC) [29] with the Bayesian Information Criterion (BIC) as score function, followed by a Maximum A Posteriori (MAP) estimation. Since missing data were present in our datasets, we used the available cases framework without the need for data imputation. We also applied some constraints to the network structure to codify the domain knowledge: clinically or biologically nonsensical relations among variables were forbidden, such as, for instance, the dependence of medical centre on patients' sex, while other dependencies were enforced, such as the dependencies of the MiToS variables and the survival from the time since onset, in accordance with the progressive nature of the disease over time [5, 12, 30].

For more details on the DBNs and a complete description of the rules set in the learning phase, see Section “3. Methods” in the Supplementary Information.

### Patient simulation

The trained ALS DBNs can be used to simulate ALS progression starting from the patients’ data at a specific visit, simulating the successive instants one at a time using the learned conditional dependencies. Since for a given node (variable) in the model in-going edges represent conditional probability dependencies from the values of its parents at the previous time-point, the state probability of the node at a certain next time-point (*t*) can be inferred using all the values of its parents at the previous time-point (*t *− 1). In this way, the ALS evolution can be step-by-step simulated and followed in terms of progression trajectories. It is worth highlighting that, when we run the model on the test set, a single starting time point is used for each subject (the first real available visit) and the system is let to evolve over time-based solely on the learned model structure and parameters (i.e., without using any other time point of the test data). In the current implementation, the tool requires as a starting point a visit with all recorded values of the variables (in other words, no missing values are allowed in the starting point visit for the test set).

### Model performance assessment

The simulation process also allows the validation of the DBN models. By comparing the simulated prognosis for each patient and the true disease progression, indeed, it is possible to assess the prediction accuracy of the learnt DBNs.

Specifically, the whole dynamic of ALS progression recorded in the training sets was used to learn the DBNs. Then, the evolution of the disease was simulated for the subjects of each test set by setting the real first recorded contact with the medical centre as the starting point and using the corresponding trained DBN to predict the progression, visit after visit, by sampling from the learned conditional probability distributions. Finally, we extracted from the so-predicted follow-ups some endpoints of interest (namely, the 4 MiToS impairments and the survival). In general, if not already recorded at the starting point visits, the impairment outcomes can occur at any time point of the simulated follow-up, while the occurrence of the simulated death event ends the simulation. We then compared the simulated time of occurrence for each outcome with the true one recorded in the patient’s real follow-up, to assess the prediction performance. To obtain probability estimates of the predicted trajectories and the corresponding outcomes’ times, a total of 100 different simulations (or repetitions) were run for each patient, each one evolving for 40-time steps or until the simulated death was reached. Each new visit at time *t* obtained through the DBN is simulated at a temporal step from the previous one that is encoded in the time between visit (TBV) variable. As per the other variables, the value of TBV(*t*) is simulated by sampling from its real distribution in the corresponding training set based on the values of its parents at time *t *− 1. The choice of simulating up to 40-time steps ensures that, for at least most of the cases, the simulated prognosis covers the mean follow-up of an ALS patient and that the survival endpoint is reached (a condition that stops the simulation).

### Statistical analysis

The continuous variables are reported as means ± SD, the categorical variables as frequencies and proportions. Kruskal–Wallis and *χ*^2^ tests at 0.01 significance level were used for assessing the equality of the distributions of the continuous and the categorical variables, respectively, in the training and independent test sets.

We evaluated the prediction accuracy of the tool over time by employing for each clinical outcome, (that is, the 4 MiToS impairments and the survival) two measures of predictive accuracy: discrimination and calibration.

*Discrimination* is the ability to discriminate between subjects at different risks, i.e., that a patient who experiences a certain clinical outcome is assigned a higher risk value by the model than a patient who will experience that outcome later. The integrated area under (AU) the receiver operating characteristic (iAU-ROC) curve is the standard measure of discrimination since it can be shown that it is equal to the C-index where 1.0 implies perfect ranking based on risk and 0.5 implies no discrimination [31]. To first evaluate the accuracy of our model over time, we computed the AU-ROC for each clinical outcome at a 3-month step from the first visit up to 96 months. The 3-month step was chosen based on the mean time between visits of both the ITIS and the IT full datasets (3.5 and 3.3 months, respectively, see Tables 1 and 2). We stopped the computation at 96 months since the percentage of deceased patients exceeded 95% in the following year. We finally calculated, for each clinical outcome, the integral of the AU-ROCs computed at the 3-month steps up to 24, 36, and 96 months.

**Table 1:** Demographic and clinical features of the ALS population included in the ITIS dataset

	Full dataset (*n* = 3940)	Training set (*n* = 3221)	Test set (*n* = 719)	*p* value
Medical centre				
Emilia-Romagna	762 (19.3%)	605 (18.8%)	157 (21.8%)	< 0.01
Maugeri Foundation	165 (4.2%)	126 (3.9%)	39 (5.4%)	
Nemo Clinical Centre	269 (6.8%)	223 (6.9%)	46 (6.4%)	
Hadassah Medical Centre	191 (4.8%)	186 (5.8%)	5 (0.7%)	
Tel Aviv Medical Centre	781 (19.8%)	633 (19.7%)	148 (20.6%)	
Piemonte and Valle d'Aosta	1772 (45.0%)	1448 (45.0%)	324 (45.1%)	
Sex				
Female	1733 (44.0%)	1418 (44.0%)	315 (43.8%)	0.90
Male	2205 (56.0%)	1801 (55.9%)	404 (56.2%)	
<NA>	2 (0.1%)	2 (0.1%)	0 (0.0%)	
Onset site				
Bulbar	1180 (29.9%)	958 (29.7%)	222 (30.9%)	0.57
Spinal	2742 (69.6%)	2245 (69.7%)	497 (69.1%)	
<NA>	18 (0.5%)	18 (0.6%)	0 (0.0%)	
Age at onset (years)	62.7 ± 11.9	62.6 ± 12.1	62.9 ± 11.2	0.98
Diagnostic delay (months)	11.9 ± 12.3	12.0 ± 12.6	11.6 ± 10.8	0.89
Time between visits (months)	3.5 ± 5.0	3.5 ± 5.0	3.4 ± 5.0	0.73
Time since onset (months)	30.6 ± 27.8	30.9 ± 28.4	29.1 ± 25.0	0.26
MiToS walking/self-care				
Experiencing impairment	2712 (68.8%)	2188 (67.9%)	524 (72.9%)	< 0.01
Not experiencing impairment	1228 (31.2%)	1033 (32.1%)	195 (27.1%)	
MiToS swallowing				
Experiencing impairment	1252 (31.8%)	1007 (31.3%)	245 (34.1%)	0.10
Not experiencing impairment	2688 (68.2%)	2214 (68.7%)	474 (65.9%)	
MiToS communication				
Experiencing impairment	829 (21.0%)	669 (20.8%)	160 (22.3%)	0.33
Not experiencing impairment	3111 (79.0%)	2552 (79.2%)	559 (77.7%)	
MiToS breathing				
Experiencing impairment	1308 (33.2%)	1056 (32.8%)	252 (35.0%)	0.20
Not experiencing impairment	2632 (66.8%)	2165 (67.2%)	467 (65.0%)	
Time to MiToS walking/self-care impairment (months)	28.4 ± 24.2	29.0 ± 25.5	26.0 ± 17.5	0.43
Time to MiToS swallowing impairment (months)	29.1 ± 20.7	29.4 ± 21.4	28.0 ± 17.7	0.89
Time to MiToS communication impairment (months)	34.9 ± 26.7	36.0 ± 27.9)	30.4 ± 19.7	0.02
Time to MiToS breathing impairment (months)	31.8 ± 27.4	32.7 ± 29.2	28.3 ± 17.7	0.34
Survival				
Censored	739 (18.8%)	595 (18.5%)	144 (20.0%)	0.28
Tracheostomised/dead	3201 (81.2%)	2626 (81.5%)	575 (80.0%)	
Time to tracheostomy/death or censoring (months)	35.7 ± 29.8	35.9 ± 30.8	34.4 ± 24.8	

Kruskal–Wallis and *χ*^2^ tests at 0.01 significance level were used for assessing the equality of the distributions of the continuous and the categorical variables, respectively, in the training and independent test sets

**Table 2:** Demographic and clinical features of the ALS population included in the IT dataset

	Full dataset (*n* = 1767)	Training set (*n* = 1504)	Test set (*n* = 263)	*p* value
Medical centre				
Emilia-Romagna	594 (33.6%)	516 (34.3%)	78 (29.7%)	< 0.01
Maugeri Foundation	123 (7.0%)	122 (8.1%)	1 (0.4%)	
Nemo Clinical Centre	209 (11.8%)	192 (12.8%)	17 (6.5%)	
Piemonte and Valle d'Aosta	841 (47.6%)	674 (44.8%)	167 (63.5%)	
Sex				
Female	800 (45.3%)	696 (46.3%)	104 (39.5%)	0.03
Male	967 (54.7%)	808 (53.7%)	159 (60.5%)	
Onset site				
Bulbar	548 (31.0%)	459 (30.5%)	89 (33.8%)	0.24
Spinal	1219 (69.0%)	1045 (69.5%)	174 (66.2%)	
Familial				
No	1607 (90.9%)	1364 (90.7%)	243 (92.4%)	0.50
Yes	116 (6.6%)	96 (6.4%)	20 (7.6%)	
< NA>	44 (2.5%)	44 (2.9%)	0 (0.0%)	
Genetics				
C9orf72	86 (4.9%)	70 (4.7%)	16 (6.1%)	< 0.01
FUS	7 (0.4%)	2 (0.1%)	5 (1.9%)	
SOD1	32 (1.8%)	29 (1.9%)	3 (1.1%)	
TARDBP	26 (1.5%)	24 (1.6%)	2 (0.8%)	
WT	1209 (68.4%)	1019 (67.8%)	237 (90.1%)	
<NA>	407 (23.0%)	360 (23.9%)	0 (0.0%)	
FTD				
No	1325 (75.0%)	1094 (72.7%)	231 (87.8%)	0.02
Yes	129 (7.3%)	97 (6.4%)	32 (12.2%)	
<NA>	313 (17.7%)	313 (20.8%)	0 (0.0%)	
Age at onset (years)	63.4 ± 11.1	63.4 ± 11.2	63.2 ± 10.9	0.70
Diagnostic delay (months)	12.7 ± 12.3	12.9 ± 12.7	11.0 ± 9.5	0.19
Time between visits (months)	3.3 ± 3.5	3.4 ± 3.7	3.0 ± 2.7	0.15
Time since onset (months)	34.6 ± 32.3	34.4 ± 31.0	35.8 ± 37.6	0.40
BMI premorbid (kg/m^2^)	26.0 ± 4.0	26.0 ± 4.1	26.0 ± 3.8	0.68
BMI at diagnosis (kg/m^2^)	24.2 ± 5.2	24.1 ± 5.3	24.4 ± 4.8	0.31
FVC at diagnosis (%)	88.4 ± 24.6	88.5 ± 24.5	88.0 ± 25.1	0.93
NIV				
Administered	726 (41.1%)	618 (41.1%)	108 (41.1%)	0.99
Not administered	1041 (58.9%)	886 (58.9%)	155 (58.9%)	
PEG				
Administered	461 (26.1%)	397 (26.4%)	64 (24.3%)	0.45
Not administered	1306 (73.9%)	1107 (73.6%)	199 (75.7%)	
Time to NIV (months)	31.9 ± 28.2	32.3 ± 28.3	29.8 ± 27.7	0.34
Time to PEG (months)	31.1 ± 22.4	31.5 ± 23.2	28.7 ± 17.1	0.36
MiToS walking/self-care				
Experiencing impairment	1226 (69.4%)	1034 (68.8%)	192 (73.0%)	0.14
Not experiencing impairment	541 (30.6%)	470 (31.2%)	71 (27.0%)	
MiToS swallowing				
Experiencing impairment	612 (34.6%)	521 (34.6%)	91 (34.6%)	0.99
Not experiencing impairment	1155 (65.4%)	983 (65.4%)	172 (65.4%)	
MiToS communication				
Experiencing impairment	371 (21.0%)	317 (21.1%)	54 (20.5%)	0.83
Not experiencing impairment	1396 (79.0%)	1187 (78.9%)	209 (79.5%)	
MiToS breathing				
Experiencing impairment	803 (45.4%)	687 (45.7%)	116 (44.1%)	0.61
Not experiencing impairment	964 (54.6%)	817 (54.3%)	147 (55.9%)	
Time to MiToS walking/self-care impairment (months)	29.7 ± 25.5	30.3 ± 25.9	26.6 ± 22.5	0.04
Time to MiToS swallowing impairment (months)	28.8 ± 19.8	28.7 ± 19.7	29.2 ± 20.4	0.78
Time to MiToS communication impairment (months)	33.1 ± 21.4	33.1 ± 21.7	33.0 ± 19.7	0.77
Time to MiToS breathing impairment (months)	32.1 ± 27.6	32.4 ± 27.6	30.5 ± 27.5	0.48
Survival				
Censored	486 (27.5%)	427 (28.4%)	59 (22.4%)	0.03
Tracheostomised/dead	1281 (72.5%)	1077 (71.6%)	204 (77.6%)	
Time to tracheostomy/death or censoring (months)	43.4 ± 33.8	43.8 ± 33.7	41.6 ± 34.4	0.17

Kruskal–Wallis and *χ*^2^ tests at 0.01 significance level were used for assessing the equality of the distributions of the continuous and the categorical variables, respectively, in the training and independent test sets

On the other hand, a good *calibration* is obtained if the model is able to predict future risk with accuracy such that the predicted probabilities closely agree with observed outcomes, i.e., the model neither underestimates or overestimates the risk. Discrimination does not affect calibration, that is, a model can perfectly rank subjects based on risk, yet being unable to predict realistic probabilities. Calibration performance was first qualitatively assessed in terms of the cumulative curve of occurrence of the real and predicted outcomes. We also performed a quantitative analysis, by computing for each outcome the number of real and simulated events (over all the repetition) occurring in the following time slices: [0,6], (6,9], (9,12], (12,18], (18,24], (24,30], (30,36], (36,96] months since the disease onset. We then compared the expected and the observed frequencies on the different time slices using as a goodness-of-fit test the *χ*^2^ test.

All analyses were conducted and figures produced using R 4.1.0 (http://www.r-project.org/) running on Windows (Windows 10).

## Results

### Demographic and clinical information of ALS patients

A total of 3940 ALS patients and 24,615 data measurements were included in the ITIS dataset (median follow-up 27 months, IQR 18–44; median number of visits equal to 5, IQR 3–8). In the IT dataset a total of 1767 ALS patients and 13,370 data measurements were included (median follow-up 34 months, IQR 23–53; median number of visits equal to 6, IQR 3–10).

We split each dataset into a training set for developing the DBN models, and a completely independent test set for validating the models. In detail, for both the datasets, we proceeded by first splitting the data into two independent random groups, in a proportion of around 80:20. Then, we verified a posteriori that the two groups were balanced, by computing the Kruskal–Wallis and *χ*^2^ tests for the continuous and the categorical variables, respectively. This procedure was repeated several times by testing different random splits. Eventually, we selected the split that provided the best stratification based on the *p* values observed across all the variables. A detailed overview of the so-obtained training and test sets is reported in Table 1 and Table 2 for the ITIS and the IT datasets, respectively.

### Dynamic Bayesian Networks of interactions among variables

**Fig. 1:**
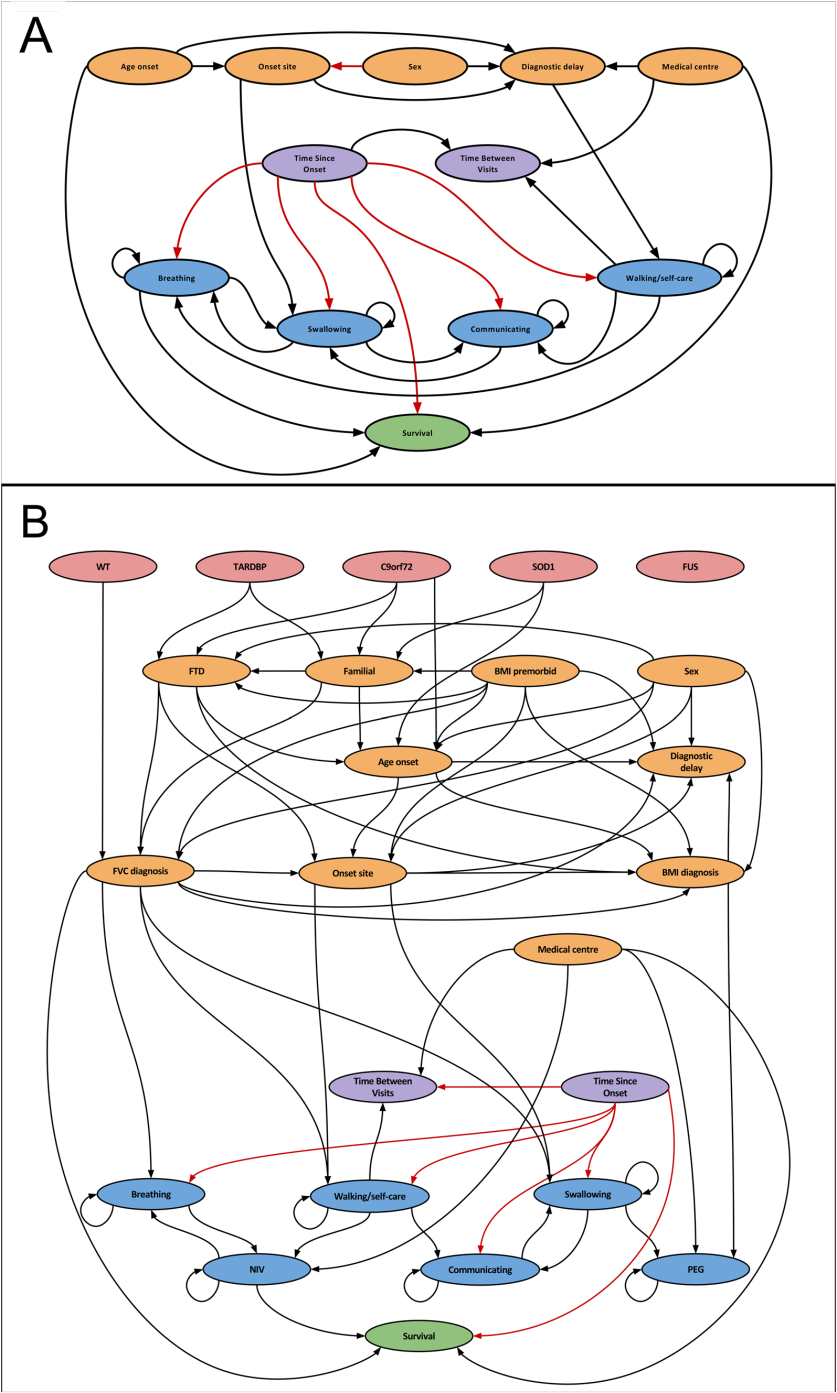
Graph representations of the **A** ITIS and **B** IT DBNs, representing the conditional dependencies among the variables over time. The loops on the four MiToS domain variables represent the dependency on the values of the same variable from the previous time-step. The red edges represent the dependencies defined as mandatory in the network learning stage

Figure 1 shows the two networks learned on the ITIS and IT training sets. By analysing their graph representations, where each node corresponds to a variable, DBNs can be used to detect inter-dependencies among variables in terms of conditional probabilities, represented as in-going edges. In inspecting the graphs, emerging dependencies previously known in the literature can serve indirectly as model structure validation.

### Model evaluation

**Fig. 2:**
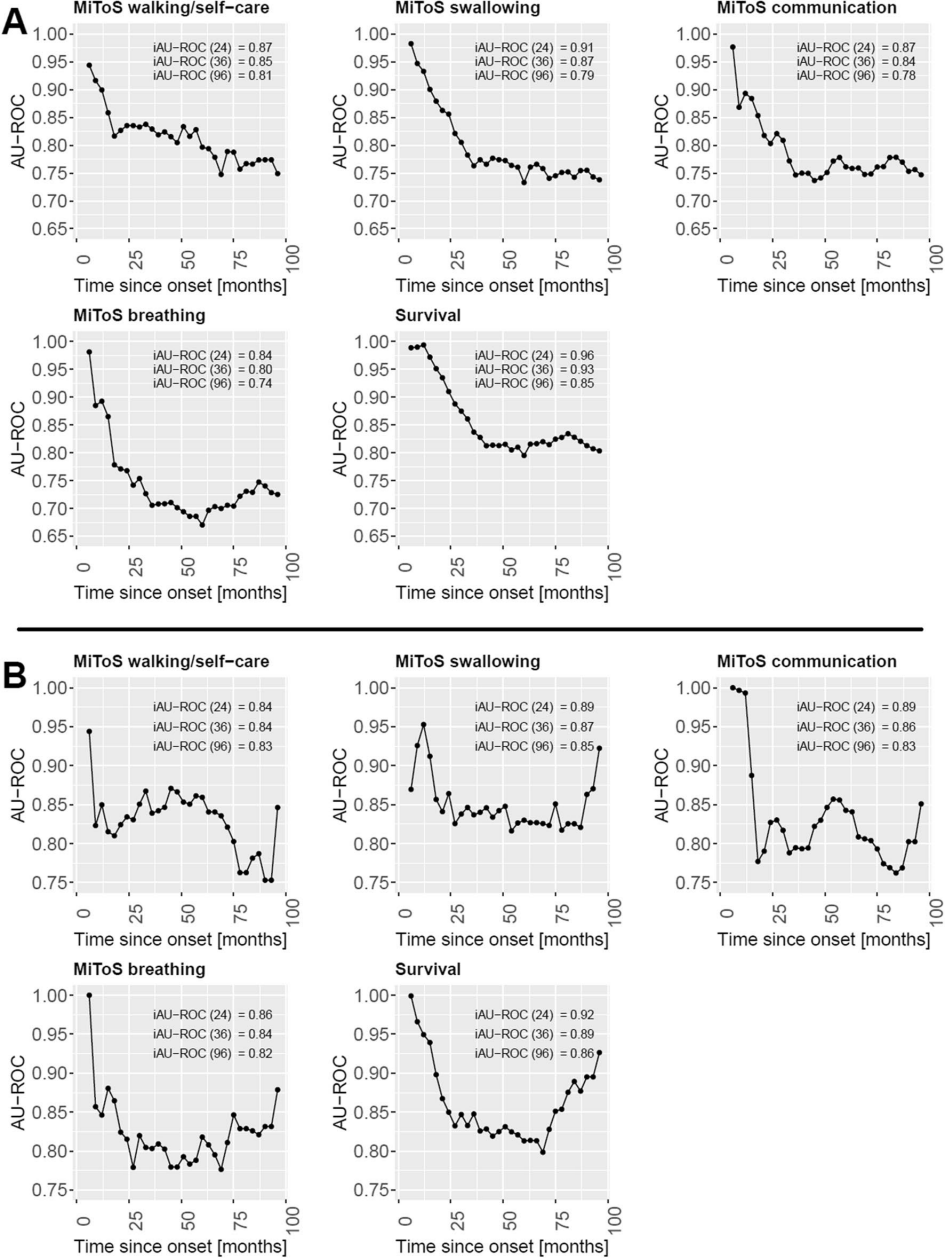
Area Under the time-dependent ROC curve (AU-ROC) for the MiToS impairments and survival on the subjects of the **A** ITIS and **B** IT test sets, computed on a 3-month time step up to 96 months since the disease onset. For each clinical outcome, the integral of the AU-ROC (iAU-ROC) computed up to 24, 36, and 96 months is also reported

The time-dependent ROC curves at various time points were computed for each predicted clinical outcome for the patients of the ITIS and IT test sets as explained in Section “Statistical analysis”. Their AU-ROC values at a 3-month step from the first visit starting from month 6 up to 96 months after the disease onset are shown in Fig. 2 for each outcome, together with the values of the iAU-ROC computed up to 24, 36, and 96 months from the disease onset.

Tables 3 and 4 report for each outcome the AU-ROC values computed on the ITIS and the IT test sets, respectively, at month 6, 9, 12, 18, 24, 30, 36 since the disease onset. We choose this time grid to explore more in detail how the models perform in the first phases of the disease. Please notice that the values of the AU-ROC at 3 months have not been computed since, up to that time point, there were no real cases of impairment/death in our test data. For each outcome and time point, in Tables 3 and 4 we also report the number of real subjects experiencing the outcome within that time in the real follow-up.

**Table 3:** Area Under the time-dependent ROC curve (AU-ROC) values computed for the MiToS impairments and survival on the subjects of the ITIS test set at 6, 9, 12, 18, 24, 30, 36 months since the disease onset

Clinical outcome	AU-ROC (number of subjects with real outcome)						
	*t* = 6	*t* = 9	*t* = 12	*t* = 18	*t* = 24	*t* = 30	*t* = 36
MiToS Walking/self-care	0.94 (*n* = 10)	0.92 (*n* = 40)	0.90 (*n* = 73)	0.82 (*n* = 162)	0.84 (*n* = 220)	0.83 (*n* = 290)	0.83 (*n* = 342)
MiToS Swallowing	0.98 (*n* = 3)	0.95 (*n* = 13)	0.93 (*n* = 27)	0.88 (*n* = 70)	0.86 (*n* = 106)	0.81 (*n* = 148)	0.76 (*n* = 186)
MiToS Communication	0.98 (*n* = 1)	0.87 (*n* = 9)	0.89 (*n* = 19)	0.85 (*n* = 45)	0.80 (*n* = 70)	0.81 (*n* = 92)	0.75 (*n* = 112)
MiToS Breathing	0.98 (*n* = 2)	0.88 (*n* = 13)	0.89 (*n* = 27)	0.78 (*n* = 79)	0.77 (*n* = 111)	0.75 (*n* = 150)	0.71 (*n* = 170)
Survival	0.99 (*n* = 12)	0.99 (*n* = 31)	0.99 (*n* = 69)	0.95 (*n* = 153)	0.91 (*n* = 242)	0.87 (*n* = 331)	0.84 (*n* = 382)

For each clinical outcome and for each time point, the number of subjects experiencing the outcome within that time in their real follow-up is reported in brackets

**Table 4:** Area Under the time-dependent ROC curve (AU-ROC) values computed for the MiToS impairments and survival on the subjects of the IT test set at 6, 9, 12, 18, 24, 30, 36 months since the disease onset

Clinical outcome	AU-ROC (number of subjects with real outcome)						
	*t* = 6	*t* = 9	*t* = 12	*t* = 18	*t* = 24	*t* = 30	*t* = 36
MiToS Walking/self-care	0.94 (*n* = 2)	0.82 (*n* = 16)	0.85 (*n* = 29)	0.81 (*n* = 67)	0.83 (*n* = 92)	0.85 (*n* = 115)	0.84 (*n* = 135)
MiToS Swallowing	0.87 (*n* = 3)	0.93 (*n* = 5)	0.95 (*n* = 8)	0.86 (*n* = 26)	0.86 (*n* = 44)	0.84 (*n* = 57)	0.84 (*n* = 71)
MiToS Communication	1.00 (*n* = 1)	1.00 (*n* = 3)	0.99 (*n* = 6)	0.78 (*n* = 13)	0.83 (*n* = 20)	0.82 (*n* = 28)	0.79 (*n* = 39)
MiToS Breathing	1.00 (*n* = 1)	0.86 (*n* = 3)	0.85 (*n* = 13)	0.86 (*n* = 30)	0.82 (*n* = 48)	0.82 (*n* = 65)	0.80 (*n* = 78)
Survival	1.00 (*n* = 4)	0.97 (*n* = 12)	0.95 (*n* = 22)	0.90 (*n* = 54)	0.85 (*n* = 90)	0.85 (*n* = 122)	0.85 (*n* = 148)

For each clinical outcome and for each time point, the number of subjects experiencing the outcome within that time in their real follow-up is reported in brackets

For both the IT and the ITIS dataset, we can observe that the models present a good ability in discriminating the risk of the subjects, with AU-ROC values over all the outcomes in the first 36 months from the disease onset that are almost always above 0.75 for the ITIS test set and above 0.80 for the IT test set.

With respect to the iAU-ROC values reported in Fig. 2, we can observe that for each outcome the accuracy of the models over time is quite good, with iAU-ROC values in the first 36 months ranging from 0.80 to 0.93 for the ITIS basic scenario model, and from 0.84 to 0.89 for the IT advanced scenario, respectively. This denotes a good concordance of the predictions with the actual disease progression and thus confirms the ability of the models to simulate clinically reliable ALS populations by using the first screening visit only.

**Fig. 3:**
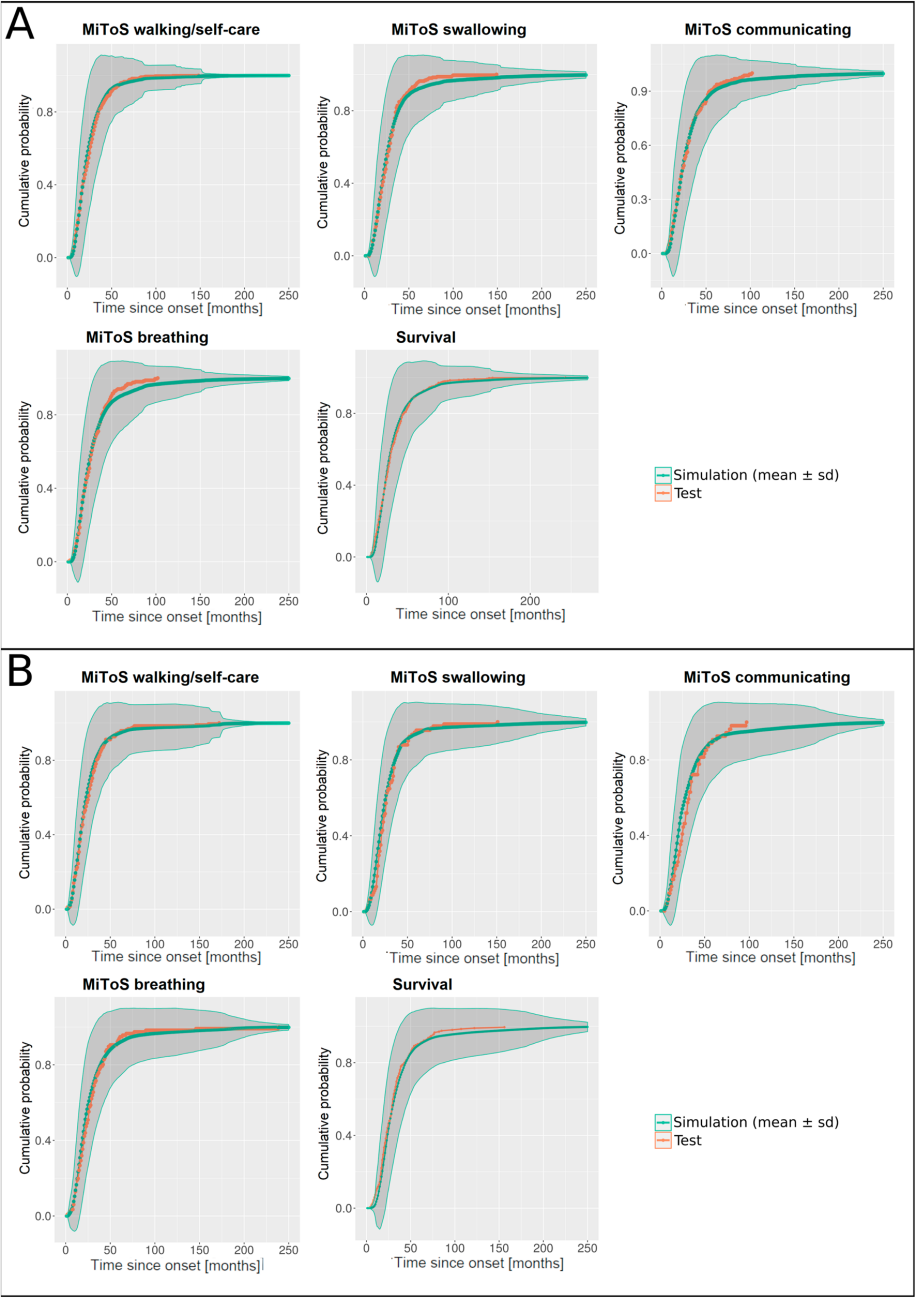
Cumulative probability of impairment in the four MiToS domains and of tracheostomy/death overtime in the **A** ITIS and **B** IT test sets (orange line) and in the simulated population (green line: mean values over population; shaded region: standard deviation), based on probabilities modelled by the DBN

Figure 3 shows the cumulative probability of the true and simulated events of MiToS impairments and tracheostomy/death overtime for the ITIS and IT test set populations. For the simulated outcomes, the confidence is reported as shaded regions. The high concordance between the predicted and actual ALS progression for both models confirms that the DBN models provide a precise simulation of survival and MiToS domain impairments.

We also quantitatively assessed the goodness of the calibration as reported in Section “Statistical analysis”. This analysis resulted in no statistically significant distributions between the expected and the observed frequencies on the different time slices (*p* values between 0.23 and 0.26 for all the outcomes), thus confirming the good calibration of both the ITIS and the IT model.

### Using the simulation tool for predicting the effect of risk factors on disease progression

The DBN model also allows patient cohort stratification, i.e., the partitioning of subjects through the identification of variables that affect the velocity of disease progression or survival. In detail, we traced how the disease course is sensitive to the change in a specific variable (risk factor), by in silico simulating ALS progression of populations with specific phenotypes at the first visit and comparing how they differentiate in terms of disease severity and/or survival time.

**Fig. 4:**
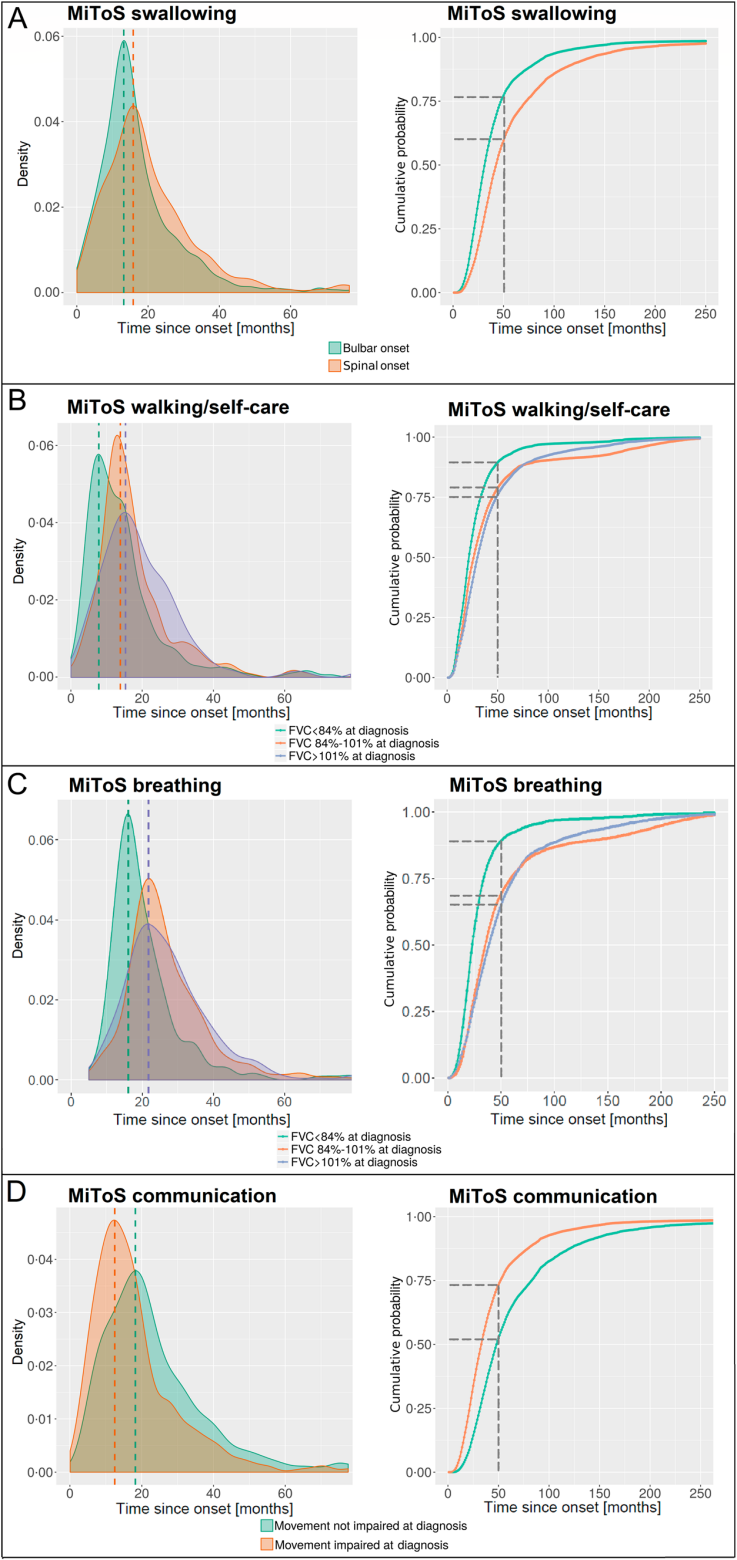
Density and cumulative probability plots of the times **A** to MiToS swallowing impairment for the patients with bulbar and spinal onset from the ITIS test set, **B** to MiToS walking/self-care impairment for the patients from the IT test set with FVC at diagnosis lower than 84%, between 84 and 101%, and higher than 101%, **C** to MiToS breathing impairment for the patients from the IT test set with FVC at diagnosis lower than 84%, between 84 and 101%, and higher than 101%, and **D** to MiToS communication impairment for the patients from the ITIS test set with and without walking/self-care impairment at the first visit. Most patients experience the impairment in correspondence with the maximum of the probability density curve (mode). For each patient, we ran 100 different simulations of the disease progression. While the density curves focus for convenience on the first months of the time span (where the distributions were more significant) the cumulative curves are shown until they reach the maximum values of 1

Figure 4A displays the effect of the *onset site* on the time to *swallowing impairment* on the patients of the ITIS test set. We split the ITIS test set into patients having a bulbar onset and patients having a spinal onset, simulated their disease evolution over time, and then finally compared their predicted times to the swallowing impairment. This analysis shows that our model is able to predict that patients with bulbar onset have a higher probability of experiencing swallowing impairment in earlier stages of the disease compared to patients with spinal onset, in keeping with previous studies [2, 5]. An effect of the onset site can also be detected by analysing the curves of cumulative probability of outcome occurrence, as reported on the right side of Fig. 4. Here, we can observe that the simulated bulbar cohort has an overall increased risk of experiencing an early swallowing impairment with respect to the spinal cohort. Marked with a grey dotted line in the plot, we can for instance observe that, at month 50 after the onset, the bulbar cohort has a cumulative probability of around 76% of having already experienced the impairment, vs. 60% of the spinal cohort.

We also studied the effect of the *FVC at diagnosis* on the time to *walking/self-care impairment* on the patients of the IT test set. We first stratified the patients of the IT test set according to their FVC at diagnosis levels into three partitions (patients with FVC at diagnosis lower than 84%, between 84 and 101%, and higher than 101%). We then simulated the ALS progression for each partition separately and compared their times to the walking/self-care impairment (see Fig. 4B). This analysis shows that the lower the FVC at diagnosis, the sooner the patients are likely to lose their walking/self-care independence. Our model predicted that the walking/self-care impairment would most likely occur at 13 months from the disease onset for the patients with an FVC value at diagnosis lower than 84%, at 18 months for those with an FVC between 84 and 101%, and at 20 months for those with an FVC higher than 101% (see density curves). These predicted values are highly concordant with the real times to impairment experienced by the patients in the IT test set (16 months for the patients with FVC lower than 84%, 18 months for those with an FVC between 84 and 101%, and 20 months for those with an FVC greater than 101%). By also looking at the curves reporting the cumulative probabilities of walking/self-care impairment given the different levels of FVC at diagnosis we can observe how having an FVC value at diagnosis lower than 84% corresponds to an overall increased risk of experiencing the impairment in the first phase of the disease. Specifically, we can observe how the cohort with lower FVC values at diagnosis has, at month 50, a risk of almost 90% of having experienced the impairment, vs. values of 79% and 75% for the other two cohorts.

On the IT test set, we also studied the effect of the *FVC at diagnosis* on the time to the *breathing impairment*. As done above, we separately simulated the patients with FVC at diagnosis lower than 84%, between 84 and 101%, and higher than 101%, obtaining the plots reported in Fig. 4C. From the density plot, we can observe how the patients with FVC at diagnosis lower than 84% are the first cohort to probabilistically experience an impairment of the breathing ability, which occurs for most of the patients around 17.5 months after the onset. The other two cohorts show a similar likely impairment at around 21.5 months. These trends also emerge from the cumulative curves, where we can observe that the risk of having a breathing impairment is much increased for the patients with FVC at diagnosis < 84% (probability at 50 months equal to 89%, vs. probability equal to 69% and 65% for the other two cohorts).

Finally, we looked at the impact of the loss of autonomy in the *walking/self-care* domain at the time of the first visit on the time to impairment in the *communication* domain. We split the ITIS test set into two partitions, separating all the patients who already had their walking/self-care impaired at the time of their first visit from the rest, and then compared the simulated time to MiToS communication impairment for the two populations. The simulation (see Fig. 4D) shows that the patients who had already experienced the walking/self-care impairment at their first visit were more likely to experience impairment in the communication domain at an earlier time point than the other patients (18 vs. 24 months after the onset). The analysis of the cumulative curves shows how, also, in this case, the risk of developing the communication impairment is generally increased over all the temporal span of progression for the cohort who already had a loss of autonomy in the walking/self-care domain. At 50 months, specifically, the risk of the already-impaired cohort is equal to 79%, vs. 52% for the non-already-impaired one.

## Discussion

We developed a probabilistic model of the progression of ALS based on DBNs using data from six different clinical centres from Italy and Israel. Being comprised of patient visits from clinical contexts and partially never investigated before, the datasets employed in this work are more representative of the general ALS population than the PRO-ACT or other clinical trials datasets.

Trained with the entire dynamics of the available data of disease progression, our models can be used to simulate and/or to predict, starting from a single time point, the entire patients’ disease progression, that can be simultaneously analysed in terms of time to the loss of independence in movement, swallowing, communication and breathing, as well as time to death.

The prediction accuracy was assessed by comparing the predicted patients’ prognosis with the real data: different performance metrics confirmed that the proposed models possess good performance in terms of both survival and domain impairment prediction. In addition, our models can also be used to stratify ALS patients into subgroups of different progression and to assess the effect of single phenotypes at diagnosis on the entire disease course.

By analysing the graphs reported in Fig. 1 and representing the networks learned on the ITIS and IT training sets, respectively, we can identify the relationships mined among the variables as well as disclose the pathways along which they influence the disease evolution. In this work, several notable inter-dependencies among variables can be identified and validated by comparison with literature results. Given a specific variable, its parents in the DBN graph can be intended as “composite biomarkers”, since the value of the variable at a certain time point can be inferred by their values at the previous one, thus extending the classic “standalone” biomarkers that have been used to date.

First, in line with expectations, we can observe that all the variables encoding the MiToS domains at a given time point, as well as NIV and PEG in the IT graph, depend on their own values at the previous time-point (graphically represented as loops). In the IT graph, NIV also depends on breathing and FVC at diagnosis (through walking/self-care), both variables related to respiratory functionality; PEG also depends on BMI at diagnosis and swallowing, both related to the initial and progressive impact of the disease on the nutrition ability.

The ITIS graph evidences that the loss of independence in breathing and in communicating at a specific time-point depends on the value of walking/self-care in the previous time-point: an impairment in walking/self-care increases the probability of experiencing an impairment in communicating and breathing in the next visits. The same relationships can be found in the IT graph as links between walking/self-care and communicating, and between walking/self-care and NIV—a variable tightly associated with the breathing ability. In both graphs swallowing and communicating appear to be interrelated, as well as swallowing and breathing in the IT graph.

In both graphs, the time between visits depends on time since onset (either enforced or detected) and on walking/self-care, indicating that the visit frequency could change based on the disease stage and its progression rate. The onset site depends on both sex (mandatory edge in ITIS, detected in IT) and age at onset, enforcing/confirming relationships known in literature: men have a greater likelihood of onset in the spinal regions, while women tend to have a higher propensity for bulbar-onset disease [2, 32, 33]; furthermore, bulbar onset is related to an older age at onset [34].

Both graphs show that survival time is dependent on, age at onset, medical centre and respiratory functionality (breathing, NIV [5, 12, 30, 35], and FVC at diagnosis [36]), besides time since onset (this latter edge was constrained in our model) [5, 12, 30]. The dependence of survival from both time since onset and respiratory function (breathing/NIV/FVC at diagnosis) is quite intuitive; the dependence from age at onset has been long known in the literature [37], being a longer survival in younger patients probably correlated to their greater neuronal reserve.

The role of the medical centre in the dependencies detected in the networks merits a closer examination. In this work we decided to aggregate different data sources: although representing a strength in terms of quantity of available data and of generalizability of the developed models, this also implies that the data may be dissimilar under different aspects (see for instance of the mean survival time and time since onset that, as reported in Tables 1 and 2, are significantly different between the IT and the ITIS datasets). Related to this, it is worth noticing that, in general, different medical centres may take charge of patients with varying disease severity, according to their specialisation level, and implement different care or screening protocols as well as policies of life support interventions. To take these facts into account, we included the variable medical centre in the dataset. Another possibility would have been to learn a different network for each centre but, since ALS is a rare disease, this would have impactfully affected the quantity of available information.

These considerations on the patients’ variability support the dependencies of the medical centre that emerged, for instance, on diagnostic delay in the ITIS graph, on NIV and PEG on the IT graph, and on the time between visits and the survival in both graphs. Since DBNs are based on joint conditional distributions dependencies, i.e., they try to explain each variable as a joint function of all its parents, the medical centre variable can, on one side, be considered as a correction factor for the bias introduced by analysing different populations together. On the other hand, the effect of this variable should be interpreted in concert with the other parents. In any case, it has to be noticed that the relationships involving other variables are detected anyway provided there is evidence in the data, and in this sense employing the medical centre variable does not result in any masking effects.

In both graphs, the relationship between onset site and swallowing may reflect the direct effect of the onset on the swallowing ability, with anticipated dysarthria and dysphagia occurrence. In addition, the direct edge from onset site to diagnostic delay validates previously reported results [38, 39]. Conversely, other studies have reported the lack of a significant difference in the diagnostic delay between bulbar- and spinal-onset patients [40, 41], leaving this relationship as an open question.

The genetic aetiology of ALS was correctly modelled in the IT graph, inferring the role on familial ALS of repeat expansion in C9orf72 and mutations in TARDBP and SOD1 [42–44]. It is also interesting to observe that there is no dependency between familiarity and FUS, in line with the fact that the latter may be affected by de novo mutation (more frequently than other genes). The graph also evidences that FTD is related to mutations in TARDBP and C9orf72 which were already associated with FTD phenotypes in previous studies [45, 46]. The influence of premorbid BMI on ALS familiarity also emerges, partially supporting the Gorges and colleagues’ study [47], which evidenced a relationship between premorbid BMI and hypothalamus atrophy.

Expected relationships among variables can also be found as indirect dependencies. For instance, the effect of the onset site on survival [2] can be identified from the following path in the ITIS graph: onset site → swallowing → breathing → survival; and from the following path on the IT graph: onset site → walking/self-care → NIV → survival. The age at onset depends on SOD1 and C9orf72 directly and on TARDBP indirectly (through the familial variable in the IT graph): interestingly, the age-related penetrance of gene mutations is currently an open question in the literature [48, 49].

Given the variables included in these models, a question could arise on the fact that the IT network does not show any direct relation between the variables FVC at diagnosis and NIV, as one might have instead expected. From the graph reported in Fig. 1, we can observe that in the IT network the NIV(t)’s direct parents are NIV(t − 1), breathing(t − 1), walking/self-care(t − 1), medical centre, and that two of these variables, namely the breathing and the walking/self-care variables, have in turn the FVC at diagnosis among their parents.

The mined relationships suggest therefore that the information provided at time *t *− 1 by the breathing and the walking/self-care variables (together with the other parents) to the NIV is strong enough for explaining the distribution of the NIV values at the next time point *t*. In this sense, we can explain the “missing” direct edge between FVC at diagnosis (which in our dataset is available only at the baseline, as a static information) and NIV as a not strong-enough relationship by itself, that can, however, be detected as mediated by the dynamic variables breathing and walking/self-care. Indeed the chain of relationships is [FVC at diagnosis] ⟶ [breathing and walking/self-care] ⟶ [NIV].

It is worth noticing that, when learning these relationships, the dynamic variables did not have the constraint of depending on themselves at the previous time point (e.g., it was not imposed for NIV(t) to depend on NIV(t − 1)), nor were these relationships forbidden. In this way, the models had the chance to learn which are the most significant parents that allow predicting the data at the next time point with the highest accuracy. Forbidding these relationships would have probably led to lower performance. Interestingly, however, the fact that these dynamic variables have other parents in addition to themselves at (t − 1) means they alone do not carry enough information to explain what will happen in the future time point.

To assess the confidence of the identified edges, a bootstrap procedure can be performed. The bootstrap technique generates different samples of a dataset and, for each sample, learns a DBN. The result is not a directed acyclic graph (DAG) and therefore it cannot be used to learn conditional probabilities, but a weighted partially DAG (WPDAG). In this latter graph, edges (*i*, *j*) weigh the number of times an edge going from node *i* to node *j* appears in a Bayesian network learned from a bootstrap sample [28]. These numbers represent a measure of the confidence in the presence of each edge. We performed this analysis on 100 bootstrap samples for both the ITIS and the IT dataset (see Section “3.3. Bootstrap-based DBN learning” in the Supplementary Information). We can observe that a number edges of the WPDAGs correspond to those constituting the DBNs learned on the whole training sets and reported in Fig. 1, thus confirming the reliability of the identified dependencies.

It has to be noticed that the fact that the DBNs are based on joint conditional probabilities means that the combination of all the parent variables together has an effect on the value of the child variable at the following time point. Therefore, it can happen that varying the value of one parent at a time does not imply a marked change in the child variable.

For instance, in the stratification studies reported in Section “Using the simulation tool for predicting the effect of risk factors on disease progression”, it can be observed how some risk factors, although discriminating the outcome, may not impact as much as one could have expected. This is the case e.g. of the site of onset with respect to the probability of experiencing a swallowing impairment (13 vs. 16 months for the bulbar vs spinal patients, respectively, marked with dotted lines in Fig. 4A). According to the DBN learned on the ITIS training set, the parents of MiToS swallowing are: MiToS breathing, onset site, time since onset (TSO), MiToS communicating, and the value of the MiToS swallowing variable itself at the previous time point. This means that the combination of all these variables together has an effect on the occurrence (or not) of an impairment in the swallowing domain at the next time point. By looking at stratified cohorts that differentiate not only on the onset site but also on others of the above-mentioned parent variables, the discriminant effect could thus be clearer. However, given the generally high number of parents for each variable in the networks, in this work we decided to limit the stratification analysis to a single variable per time, focusing on its role as a prognostic risk factor.

A possible limitation of our approach is that the proposed models can only employ discrete variables. This implies that, on one side, all continuous variables must be discretised into a finite set of levels before being processed and, on the other, the models can only predict the most probable range of each variable instead of their actual continuous values.

Another aspect concerns the management of the missing information in the data, a very common situation when handling real-world clinical data. In this work, we managed the missing data in two different ways, depending if we are in the learning phase or in the simulation/validation phase. In the learning phase, we employed an implementation of the DBNs that computes the conditional probabilities on all the combinations of the available training set data. In other words, even if some values are missing in the training samples, all the subjects’ consecutive records will still be used, limited to their available values (available-cases approach). On the other side, in the simulation/validation phase we only employed subjects with a complete first visit.

In principle, there are of course other options that can be considered. For instance, the users could first adopt an imputation procedure on the test set’s first visit patient data using state-of-the-art algorithms. As an alternative, a DBN itself can be employed for estimating the missing data, by using belief propagation for introducing knowledge received from the children variables of the network. In this way, if the value of one variable is not recorded at time t (let’s say our first visit), it can still be figured out based on the values of its children variables at time t + 1 (the second visit of the subject). However, this use of a DBN requires some care, especially if the so-imputed data are then used for prediction. This imputation may in fact be considered a sort of bias in the data since the same introduced information will be predicted through the simulation procedure. For the work presented here, therefore, we choose not to implement any imputation procedure. As a future development, we are considering extending the usability of the tool by removing the current constraint of completeness of the starting data, given of course that any embedded imputation will require a proper validation of its reliability.

Related to this, it has to be noticed how, in the current implementation of this tool, it is beneficial to have a simpler model (like the one built on the ITIS dataset) that requires the availability of only a few variables to predict the patient’s prognosis.

One important aspect that has to be taken into account when developing a tool based on DBN is the complexity of this modelling approach: in general, indeed, learning an optimal bayesian network structure is NP-hard. To address this issue, in the learning phase we adopted some constraints useful to limit the space of possible solutions, such as imposing some mandatory edges or forbidding relationships between different layers, using common sense and available know-how to drive our choices. In addition, as a heuristic to find the optimum solution, we used the Max–Min Hill-Climbing algorithm. Although reducing the learning complexity by limiting the explored space of possible networks, these choices could bring to a local minimum. On the other hand, a search of the global optimum on the entire space of possible solutions would have been computationally infeasible. Another aspect related to the constraints imposed in the learning phase is that the data available in our clinical datasets include both static and dynamic features. It was therefore necessary to appropriately define the layers and the possible dependencies among them to correctly manage these twofold temporal nature of the variables.

Despite these limitations, as far as we know our tool is the first one that, fully relying on real-world data, allows us to simulate ALS progression in a probabilistic and dynamic setting. Different from other predictive methods which allow predicting survival time or, more in general, time to some kind of event, DBNs allow modelling and predicting how all dynamic variables evolve in time and how these variables influence each other in terms of conditional dependencies. Moreover, as opposed to other models that return a punctual prediction of the time of occurrence of an outcome (e.g., [5, 6, 8]), our tool is able to simulate the whole progression trajectory of a patient from their starting visit on, thus providing a continuous estimate of the risk of experiencing multiple outcomes at the same time. From this point of view, therefore, a comparison with other methods is not straightforward.

However, we can analyse how the predictive performance of our model compares with other models built on similar data in terms of AU-ROC at a given time point or in terms of the ability to rank patients based on their risk. For sake of comparison, we employed our data to implement a Cox regression analysis with Lasso (least absolute shrinkage and selection operator) [50] considering as outcome the survival: for both the ITIS and the IT datasets, we trained a Cox-Lasso regression model on the same data used to learn the DBN models, given that such model only learns on a baseline condition that is, in our case, the first visit for each training patient. Then we assessed their predictive performance, obtaining an iAU-ROC equal to 0.74 and 0.76 on the ITIS and the IT test sets, respectively. This can be compared with the global performance of the DBNs evaluated in terms of iAU-ROC over the first 96 months from the disease onset that were found to be equal to 0.85 and 0.86 on the ITIS and IT test sets, respectively.

**Fig. 5:**
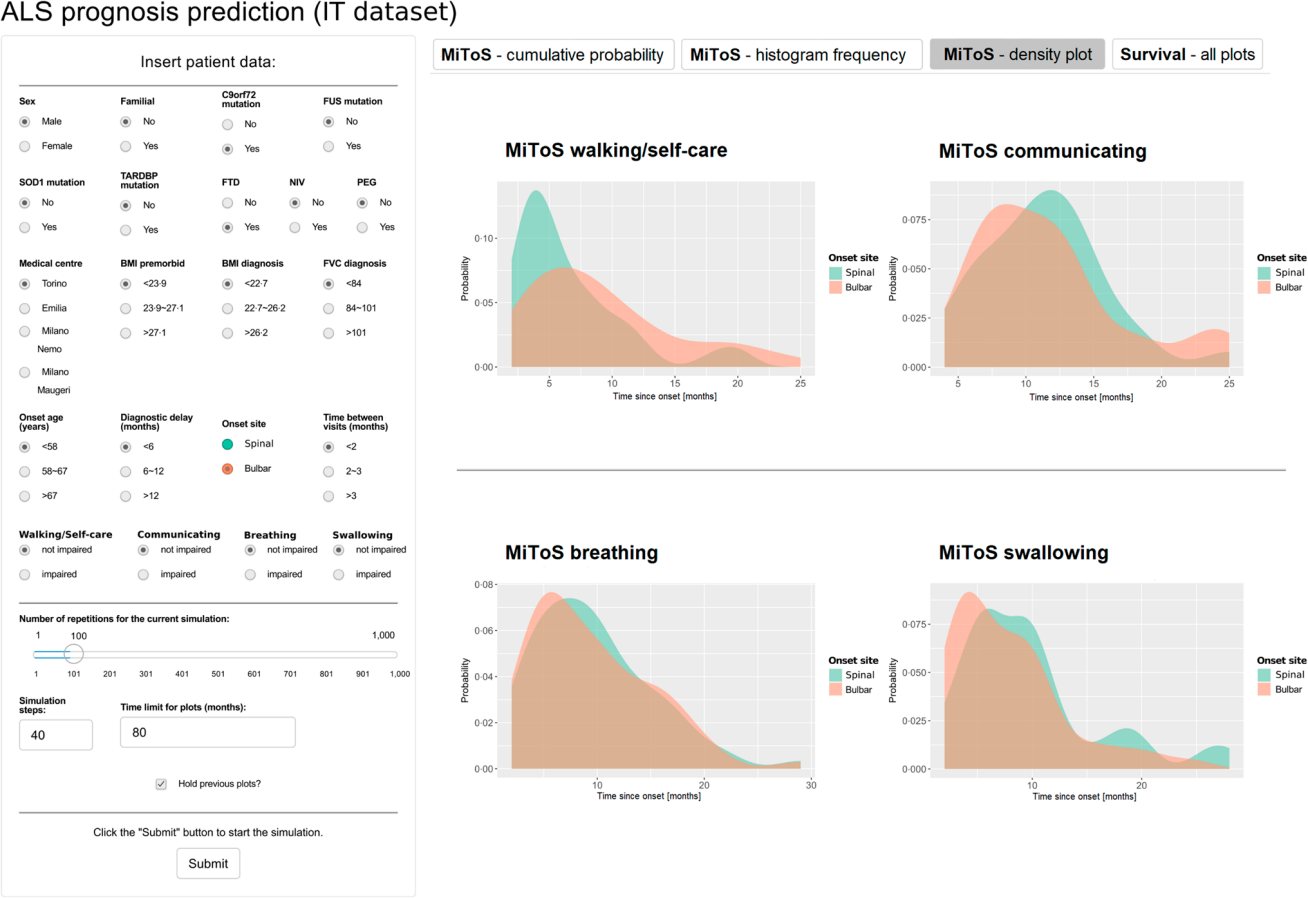
Example of single-patient ALS prognosis prediction using the web application we developed on the DBN built on the IT dataset. The figure shows the impairment probability evolution in time (months) in each of the four MiToS domains for two hypothetical patients with very similar characteristics, differing only in the onset site of the disease. Different tabs are available and allow visualisation of the probabilistic predictions of the 4 MiToS impairments and the survival over all the repetitions in terms of cumulative probability, histogram of frequencies, and density plot. The dashboard was implemented using the Shiny framework for R

Notably, we implemented a simulation dashboard based on our tool using the Shiny framework for R [51] with the aim to make it available to clinicians as an interactive web application for research use. Figure 5 shows its graphical user interface. The physician can enter on the left side of the screen the clinical data recorded during the first contact with the patient, and then start the simulation with up to 1000 repetitions (100 repetitions were used in the presented example). The plots on the right side of the screen give the probability of impairment in each of the four main MiToS domains and for survival. In our implementation, different simulations can be run sequentially, allowing the user to decide whether to keep the plots from previous simulations to be viewed alongside the plots from the last one. This way, it is possible to estimate the effect of one or more biomarkers on the ALS prognosis, simulating and assessing the impact of specific variables on risk anticipation and/or augmentation: for instance, Fig. 5 compares the effects of the spinal vs. bulbar onsets while leaving all other parameters unchanged.

An instrument able to simulate the probability of occurrence of the patients’ outcomes in the main areas of disability will have a strong impact in scheduling the allocation of the resources both at the individual and health system level, likely reducing the cost of the care by improving the provision of pharmacological and non-pharmacological therapies. The developed tool can also be used to generate in silico populations. For example, it is possible to simulate a population of subjects with bulbar onset by sampling the other variables from real data. Furthermore, a reliable model of ALS progression could potentially serve as a control group when the use of a placebo may not be appropriate or feasible or could allow a smaller control group if used in combination [11]. We are currently exploring these applications for our developed tool.

## Supplementary Information

Below is the link to the electronic supplementary material.

Supplementary file1 (PDF 461 KB)

## Data Availability

Restrictions apply to the availability of the datasets generated and/or analysed during the current study to ensure the patients' rights to privacy and anonymity and to prevent inappropriate secondary analyses. Real data or subject identity cannot be inferred in any way from the model. The data used for training and validation in this study are available upon reasonable request to the different centres involved in the study.

## Code availability

The model presented in this work has been implemented as an R code available from the corresponding author on request.
