# Supplementary material for: Predicting functional impairment trajectories in amyotrophic lateral sclerosis: a probabilistic, multifactorial model of disease progression
Source: J Neurol. 2022 Mar 10;269(7):3858–78. doi: 10.1007/s00415-022-11022-0 (PMC9217910; doi:10.1007/s00415-022-11022-0)
Supplement: Supplementary file 1 — Supplementary file1 (PDF 461 KB) [file 415_2022_11022_MOESM1_ESM.pdf]

Predicting functional impairment trajectories in amyotrophic lateral sclerosis: a probabilistic, multifactorial model of disease progression

SUPPLEMENTARY INFORMATION

Erica Tavazzi[1✦], Sebastian Daberdaku[1✦], Alessandro Zandonà[1♯], Rosario Vasta[2♯], Beatrice Nefussy[3], Christian Lunetta[4], Gabriele Mora[5], Jessica Mandrioli[6], Enrico Grisan[1,7], Claudia Tarlarini[4], Andrea Calvo[2], Cristina Moglia[2], Vivian Drory[3✤], Marc Gotkine[8✤], Adriano Chiò[2✤], and Barbara Di Camillo[1,9✤],
and for the Piemonte and Valle d'Aosta Register for ALS (PARALS)* and for the Emilia Romagna Registry for ALS (ERRALS)*

[1] University of Padova, Department of Information Engineering, Padova (Italy)
[2] University of Torino, "Rita Levi Montalcini" Department of Neuroscience, Torino (Italy)
[3] Tel Aviv Sourasky Medical Center, Tel Aviv (Israel)
[4] Centro Clinico Nemo Milano, Fondazione Serena Onlus, Milano (Italy)
[5] Istituti Clinici Scientifici Maugeri IRCCS, Milano (Italy)
[6] Azienda Ospedaliero Universitaria di Modena, Modena (Italy)
[7] London South Bank University, School of Engineering (UK)
[8] Hadassah University Hospital Medical Center, Jerusalem (Israel)
[9] University of Padova, Department of Comparative Biomedicine and Food Science, Padova (Italy)

✦ These authors contributed equally to the manuscript.
♯ These authors contributed equally to the manuscript.
✤ These authors contributed equally to the manuscript.

*Group Information: The PARALS and ERRALS members are listed at the end of the article.

Corresponding author: Barbara Di Camillo (barbara.dicamillo@unipd.it)

Table of Contents:

1. ALS staging systems 2

2. Datasets 2
2.1 Variable discretisation 2
Table 1: Quantisation levels for the variables included in the ITIS dataset. 3
Table 2: Quantisation levels for the variables included in the IT dataset. 5

3. Methods 5
3.1 Dynamic Bayesian Networks 5
3.2 Rules for learning the networks 5
3.2.1 Rules on the ITIS network 6
3.2.2 Rules on the IT network 6
3.3 Bootstrap-based DBN learning 7

References 8

1. ALS staging systems

The available ALS progression models generally rely on the ALSFRS-R staging system [1], which is a 12-item questionnaire rated on a 0-4 point scale evaluating the progression of disability in ALS patients; ALSFRS-R has been extensively used to assess treatment efficacy in clinical trials and measure disease progression [2]–[5]. Despite being a *de facto* standard, this staging system suffers from several limitations. The interpretation of an ALSFRS-R total raw score is hindered by the ambiguities of its different metric meanings for the different ALS forms [6], and by its non-linear relationship with the linear Rasch transformed measures of global function [7], [8]. The scale exhibits multi-dimensionality, thus it should not be used as a global total score [7]. The ALSFRS-R scale also suffers from the "floor-effect" and is thus unable to capture late-stage clinical changes: patients approaching the bottom of the scale appear to be "slowing down" in their worsening because it becomes increasingly difficult for them to lose further raw score points [9], [10]. Finally, there is no agreed-upon threshold at which a change in ALSFRS-R score is viewed as an important transition point in functional status [11].

To overcome these limitations, other ALS staging systems have been introduced. The King's staging system is based on disease burden as measured by the involvement of clinical regions and the presence of respiratory or nutritional failure [11], [12]. This system uses five stages, from 1 to 5, with stages 1 to 4 indicating the number of involved clinical regions (stage 1 also indicates the disease onset) and stage 5 being death. Although the King's system is not based on ALSFRS-R scores, it can be estimated from them with 92% concordance [13]. More recently, the Milano-Torino staging (MiToS) system was introduced as a novel tool to measure ALS progression [11]. This system uses six stages, from 0 to 5, with stage 0 representing symptom onset and stage 5 being death, and is based on the assessment of four functional domains (movement, communication, swallowing and breathing) assayed by the ALSFRS-R. Whether a domain is not impaired, its MiToS value is equal to 0, whereas the MiToS value is equal to 1 for the domains in which patient's independence is compromised: the MiToS score corresponds to the total number of functional domains in which the patient has lost independence. This scale was shown to be able to reliably identify relevant stages of disease in patients according to the number lost functions, to be consistent with sequential disease progression, to overcome the non-linearity and multidimensionality limitations of ALSFRS-R, and to correlate well with patients' quality of life and health service costs [14].

We employed the MiToS staging system since it tackles all the limitations of the ALSFRS-R scale while at the same time being completely derivable from the latter. The King's system, on the other hand, cannot always be fully derived from the ALSFRS-R scale, which represents a limitation. Moreover, unlike the King's staging system which summarises the clinical/anatomical spread of the disease, the MiToS system is aimed towards the distinction of functional capabilities during the spread of the disease and is able to differentiate late ALS stages in a higher resolution [15].

2. Datasets

The ITIS and IT datasets analysed in this work include static variables, which are either data collected at first visit only or time-independent covariates, and dynamic variables, that are measurements collected over subsequent visits.

In detail, static variables are: medical centre, sex, onset site, familiality, genetics, age at onset, diagnostic delay, FTD, BMI premorbid, BMI at diagnosis and FVC at diagnosis. On the other hand, time between visits (TBV), time since onset (TSO), the MiToS items, and the NIV and PEG use are dynamic variables.

2.1 Variable discretisation

In this work we employed discrete-space/discrete-time DBNs, which encode probabilistic relationships among discrete variables over a discrete number of time steps; thus, we discretised the continuous variables according to their distribution percentiles in the training sets. For all the variables of both the ITIS and the IT datasets, we set the thresholds based on the tertiles. However, for some discrete variables (such as *Time between visits*, *TBV*, in the ITIS dataset) this led to unbalanced groups since their original values were heavily distributed around specific values, while the use of quartiles corrected this bias.

For the *Time since onset (TSO)* variables, specifically, we implemented the discretization based on another requirement of the DBNs. The algorithm for DBN structure-learning relies on the assumption that the conditional probabilities distributions are time invariant. For example, breathing impairment at time (t-1) influences the breathing capability at time (t) in the same way if it is 12 months from onset or 36 months from onset. Obviously, this mathematical assumption does not hold in reality since the disease can proceed by following different patterns in its different stages. Therefore, the variable *TSO* was discretized in such a way that, within each time interval, the time-invariance assumption was verified.

Supplementary Tables 1 and 2 show the quantisation levels and the categories adopted for each variable in the ITIS and IT datasets, respectively.

**Feature**	**Level**
Medical centre	Emilia-Romagna
	Maugeri Foundation
	Nemo Clinical Centre
	Hadassah Medical Centre
	Tel Aviv Medical Centre
	Piemonte and Valle d'Aosta
Sex	Female
	Male
Onset site	Bulbar
	Spinal
Vital status	Alive
	Tracheostomised/Dead
Age at onset [years]	< 54
	[54, 62]
	[62, 69]
	> 69
Diagnostic delay [months]	< 6
	[6, 12]
	> 12
Time between visits [months]	< 1
	[1, 2]
	[2, 4]
	> 4
Time since onset [months]	< 12
	[12, 24]
	[24, 36]
	[36, 48]
	[48, 72]
	> 72
MiToS walking/self-care impairment	Yes
	No
MiToS swallowing impairment	Yes
	No
MiToS communication impairment	Yes
	No
MiToS breathing impairment	Yes
	No

Table 1: Quantisation levels for the variables included in the ITIS dataset.

**Feature**	**Level**
Medical centre	Emilia-Romagna
	Maugeri Foundation
	Nemo Clinical Centre
	Piemonte and Valle d'Aosta

Sex	Female
	Male
Site of onset	Bulbar
	Spinal
Vital status	Alive
	Tracheostomised/Dead
Familial	Yes
	No
C9orf72	Yes
	No
FUS	Yes
	No
SOD1	Yes
	No
TARDBP	Yes
	No
WT	Yes
	No
FTD	Yes
	No
Age at onset [years]	< 58
	[58, 67]
	> 67
Diagnostic delay [months]	< 6
	[6, 12]
	> 12
Time between visits [months]	< 2
	[2, 3]
	> 3
Time since onset [months]	< 11
	[11, 21]
	[21, 42]
	[42, 68]
	> 68
BMI premorbid	< 23.9
	[23.9, 27.1]
	> 27.1
BMI at diagnosis	< 22.7
	[22.7, 26.2]
	> 26.2
FVC at diagnosis	< 84
	[84, 101]
	> 101
Time to NIV [months]	< 22
	[22, 39]
	> 39
Time to PEG [months]	< 23
	[23, 37]
	> 37

MiToS walking/self-care impairment	Yes
	No
MiToS swallowing impairment	Yes
	No
MiToS communication impairment	Yes
	No
MiToS breathing impairment	Yes
	No

Table 2: Quantisation levels for the variables included in the IT dataset.

3. Methods

3.1 Dynamic Bayesian Networks

Dynamic Bayesian Networks are a generalisation of Markov decision processes and can be used to represent medical knowledge explicitly in terms of causes and effects as obtained from clinical data and domain knowledge. A DBN is defined by its structure (set of parent-children dependencies) annotated with a set of conditional probability distributions (CPDs), since each node is a probabilistic function of its parents. Nodes in a DBN are connected through a Directed Acyclic Graph. However, DBNs allow encoding cycles and feedbacks between variables when considering their relationships over different time slices. Consequently, our DBN model was developed through a two-step iterative procedure: 1) by inferring the graph topology and 2) learning the parameters of each CPD (*i.e.*, the probability that a variable assumes a specific value conditional to each possible joint assignment of values to its parents). The algorithm for DBN structure-learning relies on the following assumptions: two nodes cannot be a deterministic function of a single variable, the CPDs are time invariant, and variables are related to each other over a discrete number of time steps, called slices. For example, breathing impairment at time *t-1* influences the breathing capability at time *t*. Finally, the CPDs are usually estimated through techniques like Bayesian estimation or (regularised) maximum likelihood.

In this work, the DBN structure was inferred using the Max-Min Hill-Climbing (MMHC) algorithm [16], a greedy search-and-score method that starts with an initial graph (empty graph in our case) and searches the complete space of possible graph structures, by adding, reversing or deleting edges. The MMHC runs until a specific score (here the Bayesian Information Criterion) is maximised, or a specific number of iterations has been reached. Thus, the structure-learning phase provides the DBN topology with the highest probability of generating the training data. Subsequently, parameters of CPDs were computed through a maximum *a posteriori* estimation for each node.

The entire learning procedure was implemented in R, by using *bnstruct* [17], a package performing DBN inference on discrete and categorical data even in the presence of missing values, which is the case of our data and a common situation in the clinical context. Furthermore, *bnstruct* makes use of state-of-the-art algorithms for network learning and also provides methods for bootstrap resampling of the data and inference. *bnstruct* also allows the encoding of the domain knowledge, by applying constraints to the network topology; that can be set to forbid clinically or biologically non-sense relations among variables (see next section). These constraints also permit the exploration of only part of the solution space, reducing the computational complexity of learning the DBN, a task that is in general NP-hard. It has to be noticed that, although reducing the learning complexity, these choices could bring to a local minimum. On the other hand, a search of the global optimum on the entire space of possible solutions would have been computationally infeasible.

3.2 Rules for learning the networks

When learning the structure of the network from the training set, the following information can be provided:

1. the *mandatory edges*, i.e. the edges between variables that must be present in the network
2. the *possible edges*, *i.e.*, the edges that can be found during the learning phase. They can be defined by grouping variables in separate (disjoint) layers: then, by default, variables in a given layer *j* can depend only on variables from layers $i \leq j$. Users, however, can also allow or deny specific dependencies between layers.

3.2.1 Rules on the ITIS network

The mandatory edges set for the network on the ITIS dataset are:

- the dependency of *Onset site* from *Sex*
- the dependencies of the variables *MiToS at time t* from the variable *Time Since Onset (TSO)*
- the dependencies of the variable *Survival* from the variable *Time Since Onset (TSO)*.

The layering structure was defined as follows:

- Layer 1: Sex, Age onset
- Layer 2: Medical centre
- Layer 3: Onset site
- Layer 4: Diagnostic delay
- Layer 5: MiToS variables at time (t-1)
- Layer 6: Time Between Visits
- Layer 7: MiToS variables at time t
- Layer 8: Survival
- Layer 9: Time Since Onset

The following rules were then defined among the layers:

- Layer 1 can not depend on itself or any other layer.
- Layer 2 can not depend on itself or any other layer.
- Layer 3 can only depend on itself and layer 1.
- Layer 4 can only depend on layers 1, 2 and 3.
- Layer 5 can not depend on itself or any other layer.
- Layer 6 can only depend on layer 2 and 5.
- Layer 7 can depend on any other layer, except for itself and layers 6 and 8.
- Layer 8 can depend on any other layer, except for itself and layers 6 and 7.
- Layer 9 can not depend on itself or any other layer.

3.2.2 Rules on the IT network

The mandatory edges set for the network on the IT dataset are:

- the dependencies of the variables *MiToS at time t* from the variable *Time Since Onset (TSO)*
- the dependency of the variable *Survival* from the variable *Time Since Onset (TSO)*
- the dependency of the variable *Time Between Visits (TBV)* from the variable *Time Since Onset (TSO)*

The layering structure was defined as follows:

- Layer 1: Sex, Genetics (TARDBP, C9orf72, SOD1, FUS, or wild type WT), BMI premorbid
- Layer 2: Familiality
- Layer 3: Medical centre
- Layer 4: Age onset, FTD, Onset site, FVC diagnosis, BMI at diagnosis
- Layer 5: Diagnostic delay
- Layer 6: MiToS, NIV, PEG variables at time (t-1)
- Layer 7: Time Between Visits
- Layer 8: MiToS, NIV, PEG variables at time t
- Layer 9: Survival
- Layer 10: Time Since Onset

The following rules were then defined among the layers:

- Layer 1 can not depend on itself or any other layer.
- Layer 2 can only depend on layer 1.
- Layer 3 can not depend on itself or any other layer.
- Layer 4 can only depend on itself and layers 1 and 2.
- Layer 5 can only depend on layers 1 to 4.
- Layer 6 can not depend on itself or any other layer.

- Layer 7 can only depend on layers 3, 6 or 10.
- Layer 8 can depend on any other layer, except for itself and layers 7 and 9.
- Layer 9 can depend on any other layer, except for itself and layers 7 and 8.
- Layer 10 cannot depend on itself or any other layer.

3.3 Bootstrap-based DBN learning

In order to assess the confidence of the identified edges, a bootstrap procedure can be performed. The bootstrap technique generates different samples of a dataset and, for each sample, learns a DBN. The result is not a directed acyclic graph (DAG) and therefore it cannot be used to learn conditional probabilities, but a weighted partially DAG (WPDAG). In this latter graph, edges *(i, j)* weigh the number of times an edge going from node *i* to node *j* appears in a Bayesian network learned from a bootstrap sample [17]. These numbers represent a measure of the confidence on the presence of each edge.

We performed such analysis by employing 100 bootstrap samples. Figures 2 and 3 reported below show the resulting WPDAGs, for the ITIS and the IT datasets, respectively. For each network, the grey intensity of each edge corresponds to the number of trained bootstrapped DBNs where that edge was identified (from light grey = 1, to black = 100). With respect to the networks reported in the manuscript (manuscript's Figure 1), here the relationships between the same variable at consecutive times (e.g. the impairments in the MiToS domains) are not reported as auto-loops, but as edges from the variable at time *(t)* to the same variable at time *(t+1)*. Moreover, in this analysis, we did not impose any mandatory edge in order to assess what indication emerged from the data.

In both the networks, we can observe how a number of edges identified with high rate through the bootstrap procedure (the edges in black colour) correspond to those constituting the DBNs learned on the whole training sets and reported in the manuscript's Figure 1.

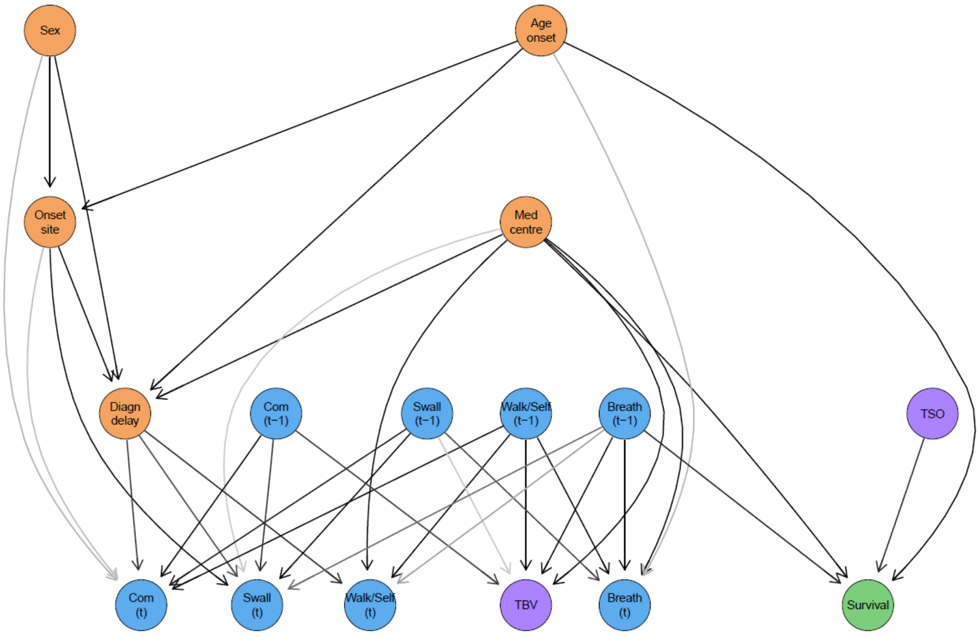

Figure 2: DBN obtained with 100 bootstrap on the ITIS training set, without mandatory edges.

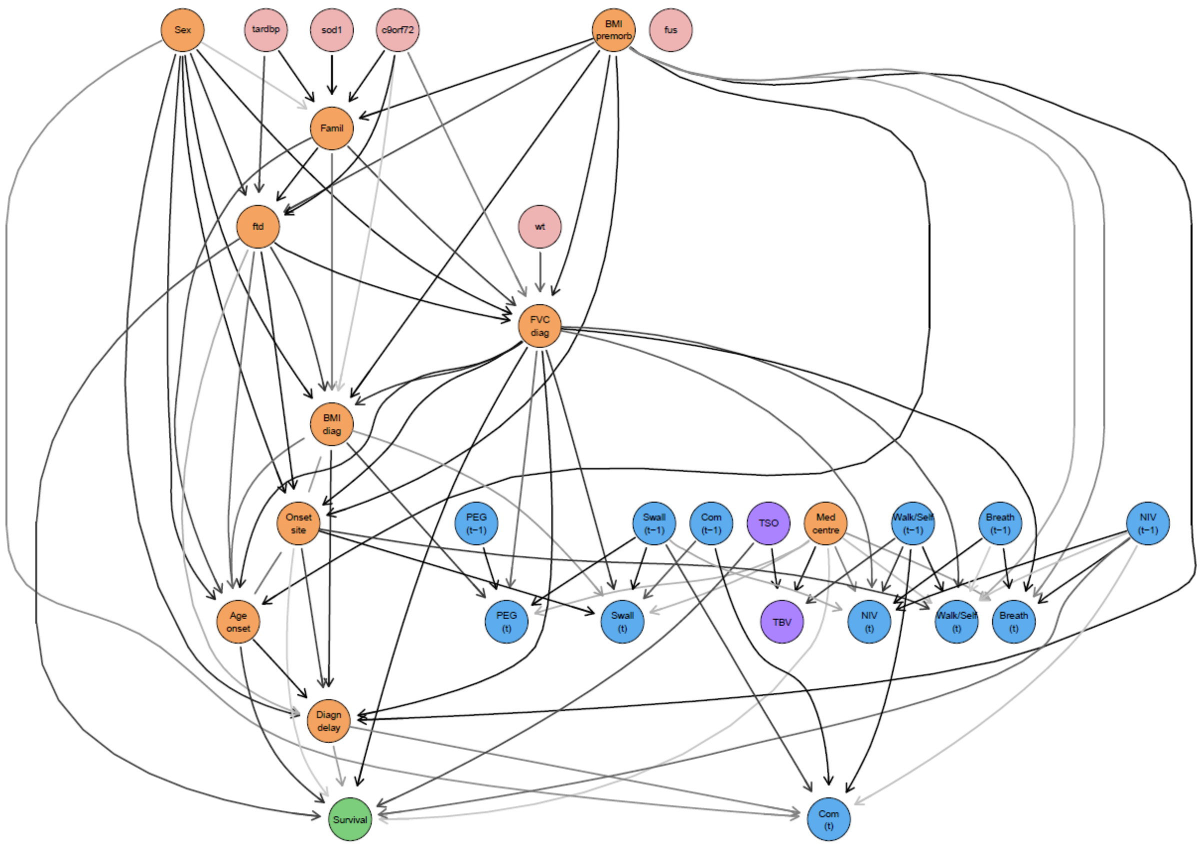

Figure 3: DBN obtained with 100 bootstrap on the IT training set, without mandatory edges.

References

[1] J. M. Cedarbaum *et al.*, "The ALSFRS-R: a revised ALS functional rating scale that incorporates assessments of respiratory function," *Journal of the Neurological Sciences*, vol. 169, no. 1–2. pp. 13–21, 1999 [Online]. Available: http://dx.doi.org/10.1016/s0022-510x(99)00210-5

[2] F. Saccà *et al.*, "A randomized controlled clinical trial of growth hormone in amyotrophic lateral sclerosis: clinical, neuroimaging, and hormonal results," *Journal of Neurology*, vol. 259, no. 1. pp. 132–138, 2012 [Online]. Available: http://dx.doi.org/10.1007/s00415-011-6146-2

[3] M. E. Cudkowicz *et al.*, "Dexpramipexole versus placebo for patients with amyotrophic lateral sclerosis (EMPOWER): a randomised, double-blind, phase 3 trial," *Lancet Neurol.*, vol. 12, no. 11, pp. 1059–1067, Nov. 2013.

[4] E. Beghi *et al.*, "Randomized double-blind placebo-controlled trial of acetyl-L-carnitine for ALS," *Amyotrophic Lateral Sclerosis and Frontotemporal Degeneration*, vol. 14, no. 5–6. pp. 397–405, 2013 [Online]. Available: http://dx.doi.org/10.3109/21678421.2013.764568

[5] T. Lenglet *et al.*, "A phase II-III trial of olesoxime in subjects with amyotrophic lateral sclerosis," *Eur. J. Neurol.*, vol. 21, no. 3, pp. 529–536, Mar. 2014.

[6] J. Mandrioli *et al.*, "Heterogeneity in ALSFRS-R decline and survival: a population-based study in Italy," *Neurol. Sci.*, vol. 36, no. 12, pp. 2243–2252, Dec. 2015.

[7] F. Franchignoni, G. Mora, A. Giordano, P. Volanti, and A. Chiò, "Evidence of multidimensionality in the ALSFRS-R Scale: a critical appraisal on its measurement properties using Rasch analysis," *J. Neurol. Neurosurg. Psychiatry*, vol. 84, no. 12, pp. 1340–1345, Dec. 2013.

[8] F. Franchignoni, J. Mandrioli, A. Giordano, S. Ferro, and ERRALS Group, "A further Rasch study confirms that ALSFRS-R does not conform to fundamental measurement requirements," *Amyotrophic Lateral Sclerosis and Frontotemporal Degeneration*, vol. 16, no. 5–6. pp. 331–337, 2015 [Online]. Available: http://dx.doi.org/10.3109/21678421.2015.1026829

[9] A. Voustianiouk *et al.*, "ALSFRS and appel ALS scores: discordance with disease progression," *Muscle*

Nerve, vol. 37, no. 5, pp. 668–672, May 2008.
[10] P. Wicks, M. P. Massagli, C. Wolf, and J. Heywood, “Measuring function in advanced ALS: validation of ALSFRS-EX extension items,” *Eur. J. Neurol.*, vol. 16, no. 3, pp. 353–359, Mar. 2009.
[11] A. Chiò, E. R. Hammond, G. Mora, V. Bonito, and G. Filippini, “Development and evaluation of a clinical staging system for amyotrophic lateral sclerosis,” *J. Neurol. Neurosurg. Psychiatry*, vol. 86, no. 1, pp. 38–44, Jan. 2015.
[12] J. C. Roche *et al.*, “A proposed staging system for amyotrophic lateral sclerosis,” *Brain*, vol. 135, no. 3. pp. 847–852, 2012 [Online]. Available: http://dx.doi.org/10.1093/brain/awr351
[13] R. Balendra *et al.*, “Use of clinical staging in amyotrophic lateral sclerosis for phase 3 clinical trials,” *J. Neurol. Neurosurg. Psychiatry*, vol. 86, no. 1, pp. 45–49, Jan. 2015.
[14] I. Tramacere *et al.*, “The MITOS system predicts long-term survival in amyotrophic lateral sclerosis,” *J. Neurol. Neurosurg. Psychiatry*, vol. 86, no. 11, pp. 1180–1185, Nov. 2015.
[15] T. Fang *et al.*, “Comparison of the King’s and MiToS staging systems for ALS,” *Amyotrophic Lateral Sclerosis and Frontotemporal Degeneration*, vol. 18, no. 3–4. pp. 227–232, 2017 [Online]. Available: http://dx.doi.org/10.1080/21678421.2016.1265565
[16] I. Tsamardinos, L. E. Brown, and C. F. Aliferis, “The max-min hill-climbing Bayesian network structure learning algorithm,” *Machine Learning*, vol. 65, no. 1. pp. 31–78, 2006 [Online]. Available: http://dx.doi.org/10.1007/s10994-006-6889-7
[17] A. Franzin, F. Sambo, and B. Di Camillo, “bnstruct: an R package for Bayesian Network structure learning in the presence of missing data,” *Bioinformatics*, vol. 33, no. 8, pp. 1250–1252, Apr. 2017.
